# Comparative transcriptome analysis reveals the resistance regulation mechanism and fungicidal activity of the fungicide phenamacril in *Fusarium oxysporum*

**DOI:** 10.1038/s41598-022-15188-5

**Published:** 2022-06-30

**Authors:** Zhitian Zheng, Huaqi Liu, Yunyong Shi, Zao Liu, Hui Teng, Sheng Deng, Lihui Wei, Yunpeng Wang, Feng Zhang

**Affiliations:** 1 School of Life Science and Food Engineering, Huaiyin Institute of Technology, Huai’an, 223003 People’s Republic of China; 2 Institute of Plant Protection, Key Lab of Food Quality and Safety of Jiangsu Province-State, Jiangsu Academy of Agricultural Sciences, Nanjing, 210014 People’s Republic of China; 3 Key Laboratory of Pesticide, College of Plant Protection, Nanjing Agricultural University, Nanjing, 210095 People’s Republic of China

**Keywords:** Drug regulation, Fungal genomics, Transcriptomics

## Abstract

*Fusarium oxysporum* (Fo) is an important species complex of soil-borne pathogenic fungi that cause vascular wilt diseases of agricultural crops and some opportunistic diseases of humans. The fungicide phenamacril has been extensively reported to have antifungal activity against *Fusarium graminearum* and *Fusarium fujikuroi*. In this study, we found that the amino acid substitutions (V151A and S418T) in Type I myosin FoMyo5 cause natural low resistance to phenamacril in the plant pathogenic Fo isolates. Therefore, we compared the transcriptomes of two phenamacril-resistant Fo isolates FoII5, Fo1st and one phenamacril-sensitive isolate Fo3_a after 1 μg/mL phenamacril treatment. Among the 2728 differentially expressed genes (DEGs), 14 DEGs involved in oxidation–reduction processes and MFS transporters, were significantly up-regulated in phenamacril-resistant isolates. On the other hand, 14 DEGs involved in ATP-dependent RNA helicase and ribosomal biogenesis related proteins, showed significantly down-regulated expression in both phenamacril-resistant and -sensitive isolates. These results indicated that phenamacril not only seriously affected the cytoskeletal protein binding and ATPase activity of sensitive isolate, but also suppressed ribosome biogenesis in all the isolates. Hence, this study helps us better understand resistance regulation mechanism and fungicidal activity of phenamacril and provide reference for the development of new fungicides to control Fo.

## Introduction

The *Fusarium oxysporum* (Fo) species complex contains many destructive fungal plant pathogens and causes vascular wilt diseases on a broad range of host plants, which involves fungal colonization of the xylem via the roots and the growing mycelium eventually causes vessel obstruction, blocks transport of water to the aerial parts of the plant^1–3^. Based on host specificity, the species complex includes more than 150 formae speciales^4^, such as Fo f. sp. *lycopersici*, Fo f. sp. *cubense*, Fo f. sp. *niveum* and so on, which infect tomato, banana, and watermelon, respectively^5–11^. In addition, some Fo also cause life-threatening invasive fusariosis in immunocompromised animals and humans^12–15^. Therefore, it is important to find an effective method to control diseases caused by Fo.

Currently, resistant cultivars, crop rotation and biocontrol using microorganisms are used to control vascular wilt disease^16–18^. In addition, the use of chemical fungicides significantly reduce Fusarium wilt. However, these Fo species typically show broad resistance to antifungal drugs and become more difficult and persistent to control^19–21^. Therefore, finding an effective antifungal compound is crucial for controlling diseases caused by Fo. Phenamacril (experimental code JS399-19; a.i. 2-cyano-3-amino-3-phenylancryic acetate) is a Fusarium-specific fungicide and shows excellent control of Fusarium head blight (FHB) and Rice bakanae disease in the field caused by *Fusarium graminearum* and *Fusarium fujikuroi*, respectively^22,23^. In our previous studies, we found that FoMyo5 motor domain substitutions (V151A and S418T) cause natural low resistance (EC_50_ value varies from 1.5 to 15 μg/mL) to fungicide phenamacril in Fo^24,25^. Thus, it is critical to elucidate the low resistance mechanism in Fo species for developing efficient control methods of phenamacril-resistant populations in the fields or immunocompromised individuals.

In *F. graminearum*, we previously reported that mutations occurred in Myosin5, encoded by FGSG_01410.1, confer to resistance to phenamacril^26^. As we know, Myosins are eukaryotic, actin-dependent ATPase motors that play important roles in actin filament bundle organization, vesicle/organelle transport, transcriptional regulation, intracellular transport, and signal transduction^27,28^. In *Fusarium asiaticum*, the mutation types of A135T, V151M, P204S, I434M, A577T, R580G/H or I581F led to low resistance to phenamacril^25^. The mutation types of S418R, I424R or A577G were responsible for moderate resistance and K216R/E, S217P/L or E420K/G/D conferred high resistance^25^. In *F. fujikuroi*, the point mutation S219P or S219 L in Myosin-5 conferred high resistance to phenamacril^23^.

Transcriptome refers to the sum of all RNA transcribed by a specific tissue or cell at a certain time or in a certain state, mainly including mRNA and non-coding RNA. Transcriptome sequencing is to study all mRNA transcribed by specific tissues or cells at a certain period^29^. It is the basis of gene function and structure research and plays an important role in understanding the development of organisms and the occurrence of diseases and exploring regulation pathways involved in pathogens response to fungicides stress^30–32^. With the development of gene sequencing technology and the reduction of sequencing cost, RNA-seq has become the main method of transcriptome research with its advantages of high throughput, high sensitivity and wide application range. RNA-seq method allows the characterization of the transcriptome even in species for which no reference genome is available. In both instances, RNA-seq reads can be assembled de novo into a transcriptome^33,34^. For example, transcriptome analysis of Fo f. sp. *niveum* after treatment with 80 μg/mL of fungicide thymol demonstrated that thymol produced reactive oxygen species (ROS) accumulation and destroyed the integrity of the cell wall and cell membrane so as to explain the mechanism of antifungal^32^.

The objectives of this paper were to study how the mutation types of V151A and S418T in FoMyo5 regulate low resistance to phenamacril, elucidate the resistance regulation mechanism of phenamacril in Fo and determine the key genes or pathways where phenamacril could inhibit the growth of Fo. For this purpose, we aligned FoMyo5 motor domains of eight Fo isolates and compared the transcriptomes of one phenamacril-sensitive isolate Fo3_a and two phenamacril-resistant isolates Fo1st and FoII5 after treated with 1 μg/mL phenamacril. The comparative transcriptome analysis of those Fo isolates could provide insight into the resistance regulation mechanism and fungicidal activity of phenamacril and provide reference for the development of new fungicides to control diseases caused by Fo.

## Results and discussion

### The activity of phenamacril against eight Fo isolates

Recently, the fungicidal activities and resistance mechanism of phenamacril have been extensively reported^23–26,35–37^. In our previous studies, we found that FoMyo5 motor domain substitutions (V151A and S418T) cause natural resistance to fungicide phenamacril in Fo^24^. However, we just tested the activity of phenamacril against six reference Fo strains before, including plant-pathogenic and -nonpathogenic or human-pathogenic strains^24^. Here, we first isolated 7 isolates of Fo from six kinds of horticultural crops suffering from Fusarium vascular wilt and one kind of cornea of patients with fungal keratitis. The majority of the isolates exhibited pink colonies and reduced aerial hyphal, except FoII5 and Fo3_a showed white colonies (see Supplementary Fig. S1). The mycelial growth rate was recorded in Table 1.

**Table 1:** Sensitivity of the *Fusarium oxysporum* isolates to phenamacril and growth rate.

Strains	EC_50_(μg/ml)^a^	Growth rate on three media (mm/day)^b^
Fo3-2	7.069 ± 0.686c	8.38 ± 0.24a
LA0	0.804 ± 0.03e	7.61 ± 0.18b
FoII5	5.512 ± 0.161d	7.46 ± 0.45bc
Fo1st	8.422 ± 0.847bc	7.38 ± 0.18bc
CAO0	11.826 ± 0.581a	7.18 ± 0.11 cd
Fo3_a	0.516 ± 0.026e	6.86 ± 0.43de
FoX-KW	9.488 ± 0.97b	6.61 ± 0.25e
FoHGKW	8.511 ± 0.615bc	6.54 ± 0.18e

^a^EC_50_ is the fungicide concentration resulting in 50% mycelial growth inhibition. Values are means ± standard deviation of three experiments, values in a column followed by the same letters are not significant difference at *P* = 0.05, Fisher’s LSD test.

^b^Growth rate was measured after incubation for 7 days. Mean and standard deviation were calculated from three replicates. The same letter indicated there was no significant difference. Different letters were used to mark statistically significant differences (*P* = 0.05).

Then we compared the susceptibility of these 7 Fo isolates and one reference Fo FoII5 to phenamacril based on mycelial growth in fungicide-amended and fungicide-free media at 28 °C. Sensitivity test showed that phenamacril exhibits stronger inhibitory activity against the human pathogenic one (Fo3_a) and Fo f. sp. *vasinfectum* (LA0). The EC_50_ values of the two isolates are 0.516 μg/mL and 0.804 μg/mL, respectively (Table 1). When treated with 2 μg/mL phenamacril, they could not grow on the PDA plates (data not show). However, the other Fo isolates collected from various hosts showed different resistance levels, the EC_50_ values varied from 5.512 to 11.826 μg/mL were recorded for FoII5, Fo3-2, Fo1st, FoHGKW, FoX-KW and CAO0 (Table 1). These six isolates of Fo demonstrated low resistance levels to fungicide phenamacril.

### Alignment analysis of the FoMyo5 motor domains in Fo

**Figure 1:**
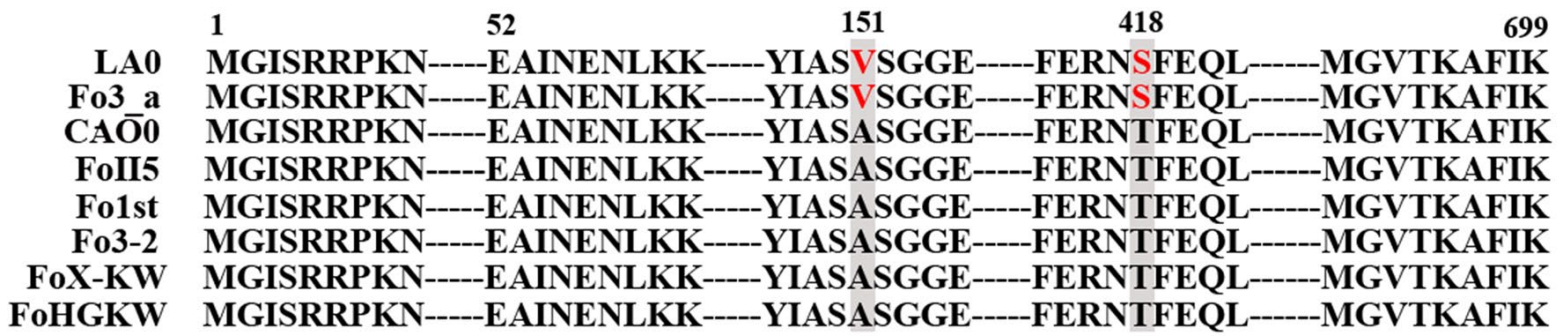
Alignment analysis of the amino acid sequences of FoMyo5 motor domains from Fo sensitive isolates Fo3_a, LA0 and natural low resistant isolates Fo3-2, FoII5, Fo1st, CAO0, FoX-KW and FoHGKW to phenamacril. The vertical boxes indicate the amino acid changes at the codon 151 and 418 that are responsible for phenamacril resistance.

To elucidate the difference of susceptibility to phenamacril among isolates Fo3_a, LA0, FoII5, Fo3-2, Fo1st, FoHGKW, FoX-KW and CAO0. We aligned these FoMyo5 motor domains using Bioedit 7.2 software and we also found two amino acid mutations at codon 151 and codon 418 in these resistant isolates (Fig. 1). These results showed that the two substitutions V151A and S418T conferred the low resistance levels to phenamacril in these field Fo isolates, which is consistent with our earlier research^24^.

### RNA-seq and de novo reference transcriptome assembly in Fo

In order to elucidate deeper insight into the resistance regulation mechanism and fungicidal activity of phenamacril in Fo, we sequenced the transcriptomes of two phenamacril-resistant isolates FoII5, Fo1st and one phenamacril-sensitive isolate Fo3_a under 1 μg/mL phenamacril treatment and 0.1 μL/mL methanol treatment condtions using the Illumina Novaseq 6000 platform. Each treatment contains three biological replicates. Raw data generated by sequencing ranged from 27.9 to 33.8 million reads per sample. After removing adaptors and low quality data, 26.7 to 32.5 million clean reads were obtained. And each library produced more than 4G clean bases with a Q20 percentage over 98% (Table 2). When we used HISAT2 software to align these clean reads to reference genome, we found that more than 70% clean reads were uniquely mapped, while the proportion of multiple mapped reads was less than 5% (Table 2).

**Table 2:** Mapping results of RNA-Seq data from phenamacril-sensitive and -resistant Fo isolates under 1 μg/mL phenamacril treatment and 0.1 μL/mL methanol treatment condtions.

Sample name^a^	Raw reads	Clean reads	Clean bases (G)	Q20 (%)	Total mapped (%)	Uniquely mapped (%)	Multiple mapped (%)
Fo1st_1	27,940,218	26,675,362	4.0	98.34	93.67	93.44	0.24
Fo1st_CK1	31,708,052	30,688,002	4.6	98.13	93.03	92.66	0.37
Fo1st_2	28,277,756	27,195,768	4.08	98.07	92.96	92.65	0.31
Fo1st_CK2	32,973,382	31,945,512	4.79	98.18	92.96	92.7	0.26
Fo1st_3	30,706,132	29,179,662	4.38	98.12	93.41	93.15	0.26
Fo1st_CK3	31,471,120	30,360,824	4.55	98.15	92.86	92.65	0.21
Fo3_a1	29,649,918	28,351,218	4.25	98.28	71.29	71.15	0.14
Fo3_aCK1	29,479,972	28,645,658	4.3	98.17	71.53	71.36	0.16
Fo3_a2	32,405,328	31,129,220	4.67	98.19	69.77	69.64	0.13
Fo3_aCK2	31,685,548	30,712,572	4.61	98.19	70.89	70.75	0.14
Fo3_a3	33,792,602	32,224,420	4.83	98.22	70.43	70.31	0.12
Fo3_aCK3	31,611,978	30,659,206	4.6	98.16	70.99	70.81	0.18
FoII5_1	31,255,586	29,083,840	4.36	98.25	89.84	85.81	4.03
FoII5_CK1	33,743,472	32,509,710	4.88	98.19	89.88	85.22	4.66
FoII5_2	31,503,714	29,148,128	4.37	98.22	87.88	82.58	5.3
FoII5_CK2	31,323,396	30,203,936	4.53	98.15	91.02	87.1	3.92
FoII5_3	28,693,422	26,966,280	4.04	98.22	91.95	88.4	3.55
FoII5_CK3	31,046,020	29,893,490	4.48	98.29	88.62	83.57	5.05

^a^Letter ‘CK’ represents the control groups treated with 0.1 μL/mL methanol and others represent the treatment groups treated with 1 μg/mL phenamacril. The number after the letters represent three biological replicates.

From the 18 high quality transcriptomes, 17,945 unigenes were obtained in total, including 970 new transcripts. These new transcripts contain transcriptional information but are not matched to the corresponding genes in the reference genome. The abundance of all the unigenes (17,945) was normalized and calculated by FPKMs (FPKM, expected number of Fragments Per Kilobase of transcript sequence per Millions base pairs sequenced) method using uniquely mapped reads. Genes with FPKMs in the interval 0–1 were considered to be present at very low levels; genes with FPKMs over 60 were considered to be expressed at a very high level. The distributions of the median expression levels of all the unigenes were up regulated in phenamacril-resistant isolates than phenamacril-sensitive isolate (see Supplementary Fig. S2). In addition, functional annotation of all the unigenes were conducted and a total of 17,945 unigenes were annotated to Gene ontology (GO), Kyotoencyclopedia of genes and genomes (KEGG) and protein protein interaction network (PPI) databases, respectively. The GO annotation indicated 9586 unigenes were categorized into 596 functional terms in 3 categories. The KEGG pathway database was used to analyze intracellular metabolic processes, and 1979 unigenes were assigned to 91 KEGG pathways.‘biosynthesis of secondary metabolites’ and ‘ribosome’ were the dominant pathways, and the proportions were 18.05%, and 10.99%, respectively. Moreover, The PPI analysis indicates that 7334 pairs of proteins encoded by the genes interacts. This high-quality transcriptome represents a valuable resource for further research on Fo isolates.

### Analysis of differential expression genes (DEGs) during phenamacril treatment

**Figure 2:**
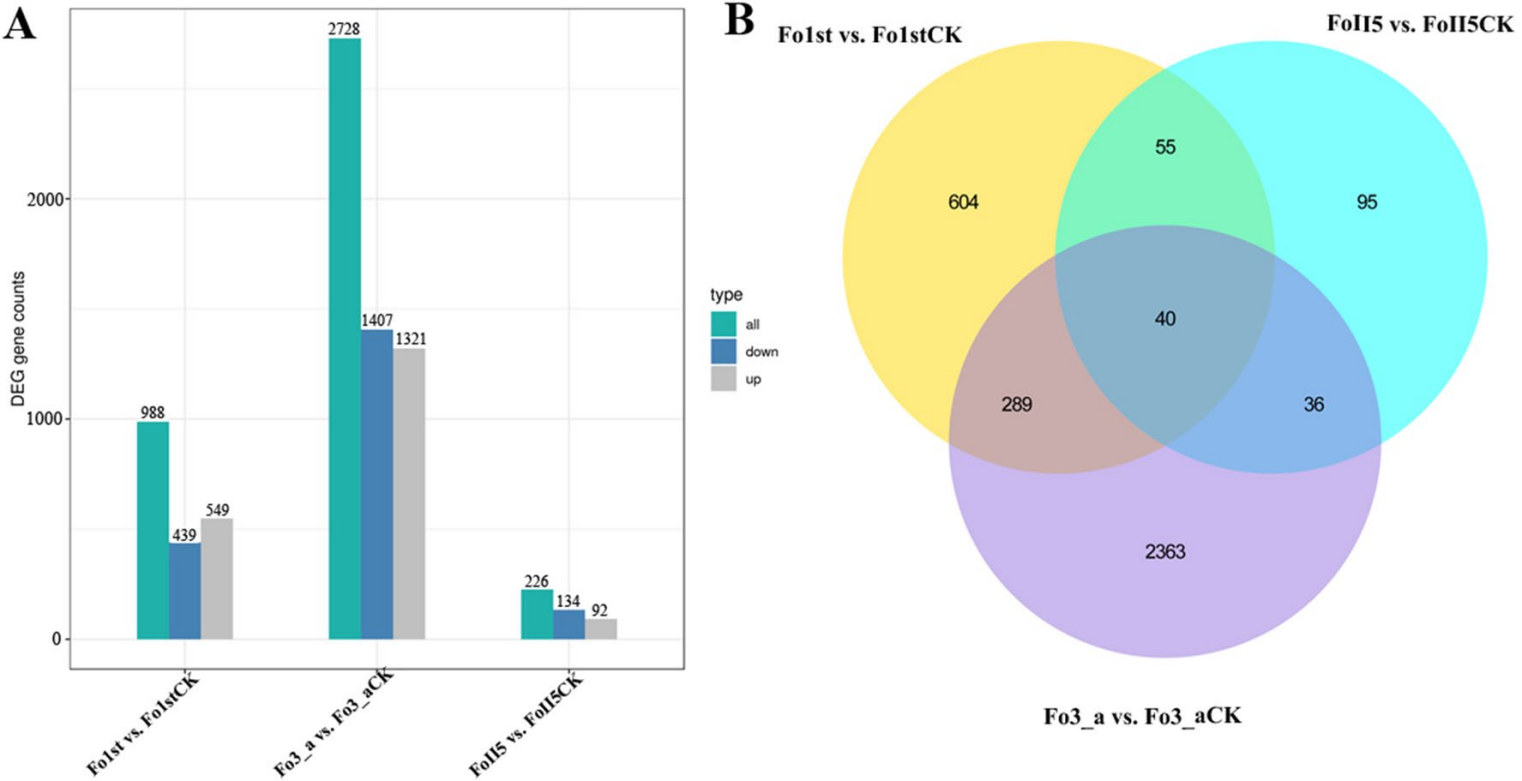
Overlapping Fo differentially expressed genes (DEGs) between phenamacril-resistant isolates Fo1st, Fo II5 and phenamacril-sensitive isolate Fo3_a during 1 μg/mL phenamacril treatment. (**A**) The number of DEGs in these Fo isolates. The blue column indicates the number of down-regulated DEGs, and the gray column indicates the number of up-regulated DEGs. (**B**) Venn’s diagram of DEGs in overlapping comparison groups datasets. The DEGs were identified by applying a threshold of an adjusted *P*-value < 0.05 and |log2 (Fold change)|> 1.‘CK’ represents the control groups treated with 0.1 μL/mL methanol and others represent the treatment groups treated with 1 μg/mL phenamacril.

One of the primary goals of the transcriptome study was to identify variations between different samples. The results indicated that these variations ranged from 226 to 2728 DEGs, based on FPKM values (Fig. 2A). When treated with 1 μg/ml phenamacril, there are 549 genes significantly up-regulated expression and 439 genes significantly down-regulated expression in phenamacril-resistant isolate Fo1st whereas there are 92 genes significantly up-regulated expression and 134 genes significantly down-regulated expression in another phenamacril-resistant isolate FoII5. However, there are as many as 1321 genes significantly up-regulated expression and 1407 genes significantly down-regulated expression in phenamacril-sensitive isolate Fo3_a (Fig. 2A). The relationships among different DEG groups from Fig. 2B were displayed as Venn diagrams, and the results indicated that 40 DEGs were identified in both phenamacril-resistant and -sensitive isolates during 1 μg/ml phenamacril treatment, of which 11 genes were significantly up-regulated and 29 genes were significantly down-regulated. In addition, a total of 55 DEGs were identified in the two phenamacril-resistant isolates but not in the phenamacril-sensitive isolate, of which 29 genes were significantly up-regulated and 26 genes were significantly down-regulated. Because these two resistant isolates are different Fo formae speciales with different genetic backgrounds, when we treated the two isolates with phenamacril, the numbers of DEGs were significantly different. However, we found that 95 DEGs of the two isolates were identical in response to phenamacril. By further studying the function of the 95 genes, we might find the mechanism of regulating resistance of Fo to phenamacril.

### Clustering Analysis of DEGs during phenamacril treatment

From the transcriptomes of Fo, large numbers of DEGs under 1 μg/mL phenamacril treatment and 0.1 μL/mL methanol treatment conditions were identified in phenamacril-sensitive and-resistant isolates. To observe the gene expression patterns, we performed hierarchical clustering of all the DEGs (3482 genes) based on the log_2_ (FPKM + 1) for the 6 treatments. From the upper and lower part of heatmap we can see, there many of the genes that are differentially expressed in the sensitive isolate Fo3_a are different from the resistant isolates Fo1st and FoII5 after phenamacril treatment. (see Supplementary Fig. S3).

### GO classification of DEGs during phenamacril treatment

**Figure 3:**
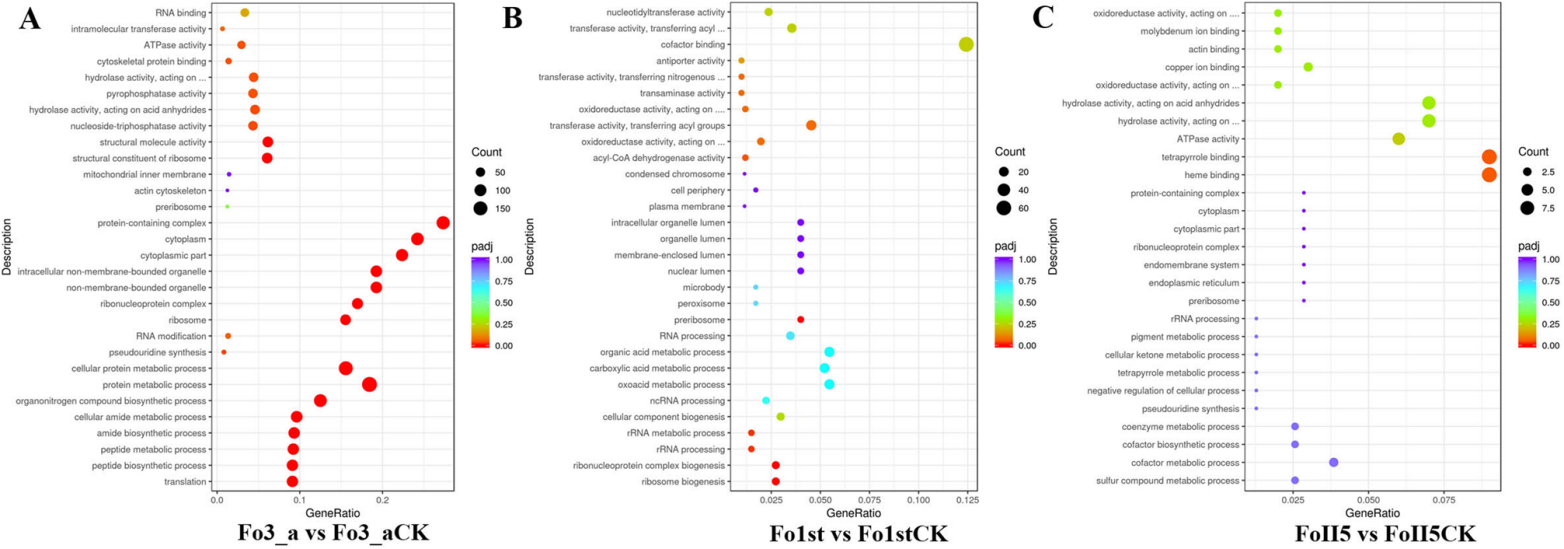
GO enrichment analysis of DEGs in all comparison groups. The top 30 most enriched GO categories were shown in a scatter diagram in the comparison groups (**A**) Fo3_a vs Fo3_aCK and (**B**) Fo1st vs Fo1stCK (**C**) FoII5 vs FoII5CK. The abscissa represents the ratio of the number of differential genes annotated on GO term to the total number of differential genes and the ordinate represents GO term. The size of small dots represents the number of genes annotated to GO term, the color from red to purple represents the significance of enrichment, and p_adj_ < 0.05 is used as the threshold of significance enrichment for GO enrichment analysis.

To classify the functions of the predicted Fo DEGs, We performed GO enrichment analysis, which is an internationally standardised gene functional classification system in biological process, cellular component and molecular function. The DEGs were assigned to 596 functional terms by enrichment analysis of GO assignments and we chose 24 functional terms based on the significant degree of enrichment analysis from high to low for study. When treated with phenamacril, In total 6 and 2 GO terms could be assigned to resistant isolates Fo1st and FoII5, respectively. In particular, ribosome biogenesis (GO:0042254, 11DEGs), ribonucleoprotein complex biogenesis (GO:0022613, 11DEGs), rRNA processing (GO:006364, 6DEGs), rRNA metabolic process (GO:0016072, 6DEGs), preribosome (GO:0030684, 7DEGs) and acyl-CoA dehydrogenase activity (GO:0003995, 6DEGs) were significantly enriched in Fo1st compared with Fo1stCK (CK represents the control groups), and heme binding (GO:0020037, 9DEGs) and tetrapyrrole binding (GO:0046906, 9DEGs) were the significantly enriched terms in FoII5 compared with FoII5CK (Fig. 3). However, there are 24 functional groups containing 2033 DEGs were enriched in sensitive isolate Fo3_a compared with Fo3_aCK, which involved in peptide biosynthetic and metabolic process, ribosome and non-membrane-bounded organelle, cytoskeletal protein binding and ATPase activity and so on (Fig. 3). Interestingly, as many as 72 DEGs, which involved in structural molecule activity (GO:0005198, 75 DEGs), were significantly down-regulated. And the cytoskeletal protein binding (GO:0008092, 17DEGs) and ATPase activity (GO:0016887, 36DEGs) were seriously affected in sensitive isolate after treated with phenamacril. These results are in accordance with the previous studies in *F. graminearum*, which revealed that phenamacril binds to FgMyoI or inhibits ATPase activity of FgMyoI motor domain and thereby reduces the stability of actin cytoskeleton^37^.

### KEGG pathway analysis of DEGs during phenamacril treatment

**Figure 4:**
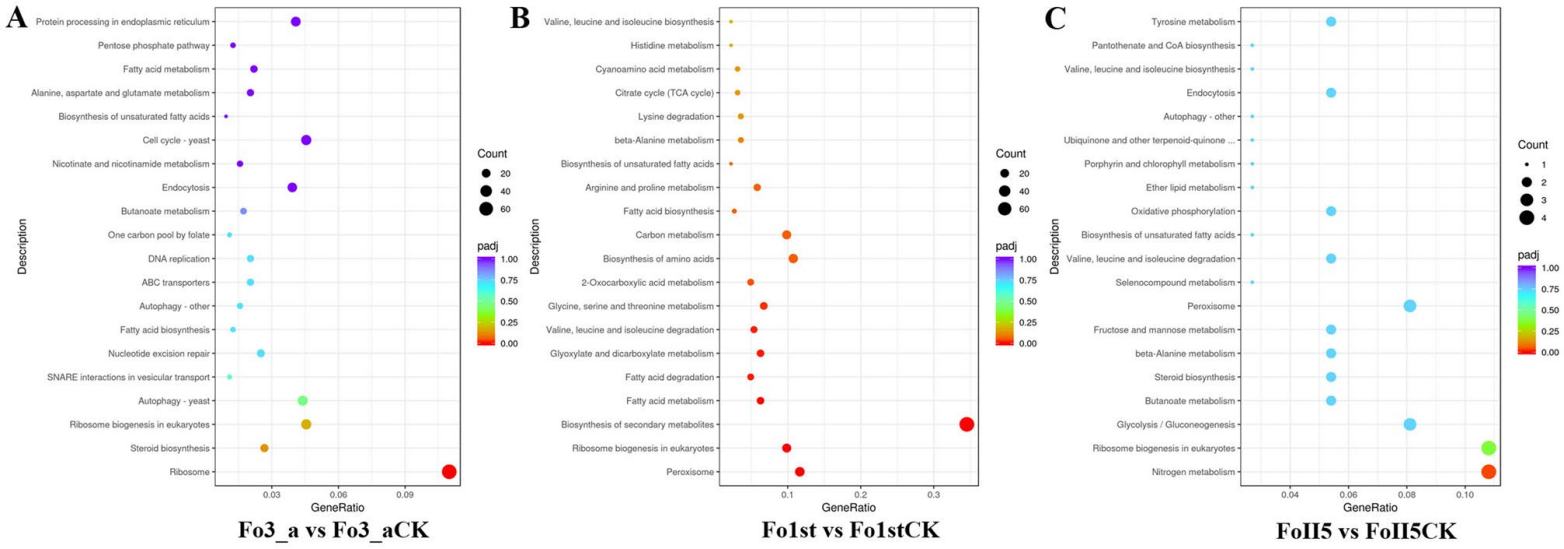
KEGG enrichment analysis of DEGs in all comparison groups. The top 20 most enriched KEGG pathways were shown in a scatter diagram in the comparison groups (**A**) Fo3_a vs Fo3_aCK and (**B**) Fo1st vs Fo1stCK (**C**) FoII5 vs FoII5CK. The abscissa represents the ratio of the number of differential genes annotated on KEGG pathways to the total number of differential genes and the ordinate represents KEGG pathways. The size of small dots represents the number of genes annotated to KEGG pathways, the color from red to purple represents the significance of enrichment, and p_adj_ < 0.05 is used as the threshold of significance enrichment for KEGG enrichment analysis.

To investigate the major biological pathways of the DEGs, we aligned all DEGs to KEGG pathways. In the Fo3_a vs. Fo3_aCK group, 91 KEGG metabolic pathways were identified and only the ribosome (70 DEGs) was the most significantly enriched pathways (p_adj_ < 0.05). Moreover, a total of 70 DEGs were all significantly down-regulated. In the Fo1st vs. Fo1stCK group, 76 KEGG metabolic pathways were identified and Biosynthesis of secondary metabolites (77 DEGs), Peroxisome (26 DEGs), Ribosome biogenesis in eukaryotes (22 DEGs), Fatty acid metabolism and degradation (25 DEGs), Glyoxylate and dicarboxylate metabolism (14 DEGs), Valine, leucine and isoleucine degradation (12 DEGs), Glycine, serine and threonine metabolism (15 DEGs), 2-Oxocarboxylic acid metabolism (11 DEGs) were the highly enriched pathways (p_adj_ < 0.05). In the FoII5 vs. FoII5CK group, 50 KEGG metabolic pathways were identified and only Nitrogen metabolism (4 DEGs) were the highly enriched pathways (p_adj_ < 0.05) (Fig. 4). However, a total of 44 identical KEGG pathways were annotated in both the two kinds of resistant isolates after treated with phenamacril, including Glycine, serine and threonine metabolism, Valine, leucine and isoleucine degradation, Fatty acid metabolism or degradation, Carbon metabolism, Nitrogen metabolism, etc (see Supplementary Table S3). These results demonstrate that the resistant isolates enhanced phenamacril tolerance through amino acid, carbon, nitrogen and fatty metabolism and degradation. In particular, phenamacril inhibited the ribosome biogenesis in eukaryotes in all the isolates (Fig. 4).

### Protein protein interaction network (PPI) analysis of DEGs during phenamacril treatment

In order to find the interacting proteins of DEGs, we conducted the analysis of PPI using STRING protein interaction database in this paper. In the Fo3_a vs. Fo3_aCK group, we found a total of 7334 DEGs with interacting proteins, in which 6228 DEGs were down-regulated and 615 DEG were up-regulated. In addition, in the Fo1st vs. Fo1stCK and FoII5 vs. FoII5CK groups, there are 2773 and 254 DEGs with interacting proteins, respectively. Interestingly, we found that FoMyo5 (Gene ID: 42025582) was only up-regulated in the Fo3_a vs. Fo3_aCK group but not in the Fo1st vs. Fo1stCK and FoII5 vs. FoII5CK groups. In addition, the interacting protein of FoMyo5, cortactin (Gene id: 42025702) and the proteins (fimbrin, actin-like protein 3, actin cytoskeleton regulator complex protein END3 and actin-like protein 2) that interacts with cortactin were all up-regulated expression ranged from 1.38 to 2.83-fold in sensitive isolate Fo3_a after treated with 1 μg/mL phenamacril (see Supplementary Table S2). These actin cytoskeleton related proteins are required for generation, maintenance, and turnover of actin filaments, contribute to ATP-loaded and rapid filament assembly, which play important roles in vesicle/organelle transport, cell polarization, transcriptional regulation, intracellular transport, and signal transduction^27,38–41^. Therefore, phenamacril might stimulated the transcription of *FoMyo5* and actin cytoskeleton related genes to maintain stability of of mycelium in the phenamacril-sensitive isolate.

### Analyzing the DEGs involved in fungicidal activity of phenamacril in Fo

In the previous studies, crystal structure of phenamacril-bound *F. graminearum* myosin I suggesting that fungicidal activity of phenamacril results from the inhibition of the ATPase activity of the myosin I^36,37^. In this paper, we found 40 DEGs were significantly expressed in both phenamacril-resistant and -sensitive isolates during phenamacril treatment (Fig. 2B). Interestingly, 14 DEGs with annotation were all down-regulated by phenamacril and these genes encoded some enzymes and proteins involved in RNA metabolism and cell membrane biosynthesis, such as ATP-dependent RNA helicase, C-8 sterol isomerase and so on (Table 3). As we know, RNA helicases are ubiquitous, highly conserved enzymes that bind or remodel RNA or RNA–protein complexes in an ATP-dependent fashion. These proteins are widely distributed in all three kingdoms of life and are associated with all biological processes involving RNA metabolism, including transcription, splicing, RNA transport, ribosome biogenesis, RNA editing, translation, and RNA decay^42–45^. Many RNA helicases are essential for viability, and a growing number of these enzymes are known to play major regulatory roles in cells^46^. The largest family of RNA helicases is the DEAD box protein family, which were shown to be involved in the ATPase and helicase activities and in their regulation^47^. In the yeast *Saccharomyces cerevisiae*, 14 DEAD box proteins were shown to be required for ribosome biogenesis and rRNA maturation^48^. Moreover, several DEAD box proteins have been shown to participate in several distinct pathways^49^. In this study, 7 DEAD box proteins (ATP-dependent RNA helicase DBP2, DBP3, DBP9, DBP10, DED1, ATP-dependent rRNA helicase RRP3, SPB4) were all down-regulated in both phenamacril-resistant and -sensitive isolates during phenamacril treatment (Table 3). In addition, 50S ribosomal protein L24e, ribosome biogenesis protein n, nucleolin and nucleolar GTP-binding protein, that are related to ribosome biogenesis^50–54^, were also down-regulated in both phenamacril-resistant and -sensitive isolates during phenamacril treatment (Table 3). These results indicated that phenamacril not only inhibits the ATPase activity of the FoMyo5, but also interferes RNA metabolism and suppresses ribosome biogenesis, thus affect protein synthesis.

**Table 3:** The DEGs involved in fungicidal activity of phenamacril in Fo.

Gene id	Gene name	Fold change (log_2_ ratio)^a^			Annotation
		FoII5 vs. FoII5CK	Fo1st vs. Fo1stCK	Fo3_a vs. Fo3_aCK	
42037078	FOIG_11903	−2.33	−3.85	−2.94	ATP-dependent RNA helicase DBP10
42034116	FOIG_08941	−2.56	−4.55	−2.63	ATP-dependent RNA helicase DBP3
42029194	FOIG_04019	−2.94	−6.25	−2.63	ATP-dependent rRNA helicase RRP3
42035835	FOIG_10660	−2.32	−3.85	−2.94	ATP-dependent RNA helicase DBP9
42029337	FOIG_04162	−2.63	−4	−1.54	ATP-dependent RNA helicase DBP2
42032836	FOIG_07661	−2.70	−3.23	−1.45	ATP-dependent rRNA helicase SPB4
42030962	FOIG_05787	−2.38	−2.17	−1.22	ATP-dependent RNA helicase DED1
42030757	FOIG_05582	−2.35	−3.61	−2.86	50S ribosomal protein L24e
42026067	FOIG_00892	−2.30	−3.46	−1.50	nucleolin
42033276	FOIG_08101	−3.36	−5	−2.75	nucleolar GTP-binding protein
42025910	FOIG_00735	−2.42	−2.92	−2.62	ribosome biogenesis protein n
42028022	FOIG_02847	−1.85	−1.84	−3.76	C-8 sterol isomerase
42034103	FOIG_08928	−1.85	−2.08	−1.88	pseudouridylate synthase
42029667	FOIG_04492	−1.89	−1.94	−1.62	PTH2 family peptidyl-tRNA hydrolase

^a^‘CK’ represents the control groups treated with 0.1 μL/mL methanol and others represent the treatment groups treated with 1 μg/mL phenamacril.

Phenamacril also suppressed the gene expressions of pseudouridylate synthase, PTH2 family peptidyl-tRNA hydrolase and C-8 sterol isomerase (Table 3). Some research showed that peptidyl-tRNA hydrolase played its critical role in protein biosynthesis and sterol C-8 isomerase played an essential regulation role in the ergosterol biosynthesis of *Saccharomyces cerevisiae*^55–57^. In particular, RNA pseudouridylate synthase was studied as novel drug target to cure Malaria caused by *Plasmodium Falciparum*^58,59^. Therefore, further research on how phenamacril inhibited the 14 functional genes, would help us to develop new fungicides to control Fo.

### Analyzing the DEGs involved in resistance regulation of phenamacril in Fo

To screen for unique DEGs involved in the resistance regulation process, we compared the transcriptome of phenamacril-resistant isolates Fo1st and FoII5 with phenamacril-sensitive isolate Fo3_a during phenamacril treatment. When treated with phenamacril, 29 DEGs were significantly up-regulated and 26 DEGs were significantly down-regulated in both the phenamacril-resistant isolates Fo1st and FoII5. However, these genes were not significantly expressed in phenamacril-sensitive isolate. In particular, 14 up-regulated DEGs with annotation in both the phenamacril-resistant isolates were linked to oxidation–reduction processes, including nitric oxide dioxygenase, nitrate reductase and MFS transporters (Table 4). Nitric oxide dioxygenase metabolize nitric oxide (NO) to nitrate by consuming NADPH and protect bacteria and fungi from NO-mediated damage and growth inhibition^60^. In fungi, the electron donor for nitrate reductase, nitrite reductase, 2,4-dienoyl-CoA reductase is NADPH^61–64^. Since phenamacril seriously affected ATPase activity, these NADPH-associated activities could produce more energy (such as ATP) to maintain mycelial growth. Researchers have reported that inhibitors reduce the catalytic activity of 4-Aminobutyrate aminotransferase^65^. In yeast, alcohol dehydrogenase plays an important role in the conversion of alcohols to aldehydes or ketones^66^. During cellular metabolism, the triacylglycerol in the form of stored energy rapidly can be rapidly metabolized during times of low carbohydrate availability or during heightened metabolic demand (cold-stress) for survival^67^. Pyridoxinamine 5'-phosphate oxidases that bind flavin mononucleotide (FMN) are an important class of enzymes that play a central role in cell metabolism^68^. Studies have shown that the utilization of primary amines as nitrogen source by yeasts and moulds is the possession of an amine oxidase^69^. Through these studies, we conclude that the up-regulated expression of nitric oxide dioxygenase, nitrate reductase, nitrite reductase, 2,4-dienoyl-CoA reductase, 4-Aminobutyrate aminotransferase, alcohol dehydrogenase, triacylglycerol lipase, pyridoxinamine 5'-phosphate oxidase, primary amines and MFS transporters enhanced the resistance of phenamacril-resistant isolates to phenamacril (Table 4).

**Table 4:** The DEGs involved in resistance regulation of phenamacril in Fo.

Gene id	Gene_name	Annotation	Fold change (log_2_ ratio)		
			Fo1st vs. Fo1stCK	FoII5 vs. FoII5CK	Fo3_a vs. Fo3_aCK^**a**^
42029749	FOIG_04574	Nitric oxide dioxygenase	6.47	9.96	NA
42031140	FOIG_05965	Uroporphyrin-III C-methyltransferase/ precorrin-2 dehydrogenase/ sirohydrochlorin ferrochelatase	3.42	6.08	1.76
42035331	FOIG_10156	Triacylglycerol lipase	10.78	3.38	NA
42031672	FOIG_06497	Alcohol dehydrogenase	3.26	7.68	NA
42034308	FOIG_09133	MFS transporter, SP family, general alpha glucoside:H + symporter	21.08	6.65	NA
42026895	FOIG_01720	Pyridoxamine 5'-phosphate oxidase	6.63	3.91	NA
42031895	FOIG_06720	Nitrate reductase [NADPH]	6.11	6.77	1.51
42027369	FOIG_02194	Nitrite reductase [NADPH]	3.36	4.04	NA
42039770	FOIG_14595	Primary-amine oxidase	6.11	3.11	NA
42029419	FOIG_04244	MFS transporter, SHS family, lactate transporter	10.51	4.88	−3.03
42026345	FOIG_01170	2,4-dienoyl-CoA reductase [NADPH]	3.77	2.81	−1.92
42027069	FOIG_01894	Acetyl-CoA hydrolase	1.66	1.79	NA
42030291	FOIG_05116	4-Aminobutyrate aminotransferase	4.53	3.30	−4.24
42031645	FOIG_06470	Dihydroxy-acid dehydratase mitochondrial	3.79	3.45	−3.64
42027968	FOIG_02793	Succinate dehydrogenase (ubiquinone) flavoprotein subunit	−2.43	−1.89	2.26
42027532	FOIG_02357	Elongation factor 3	−5.03	−3.45	NA
42031693	FOIG_06518	ABC transporter ATP-binding protein ARB1	−3.10	−2.08	NA
42032779	FOIG_07604	Chitinase	−2.06	−1.57	NA
42029731	FOIG_04556	Biphenyl-2,3-diol 1,2-dioxygenase	−3.79	−3.13	NA
42030858	FOIG_05683	Peptidyl-prolyl cis–trans isomerase NIMA-interacting 4	−1.71	−2.13	1.50

^a^NA represents no significant difference or no expression level of genes was detected. The fold change value is represented by the log2 ratio. ‘CK’ represents the control groups treated with 0.1 μL/mL methanol and others represent the treatment groups treated with 1 μg/mL phenamacril.

On the other hand, 26 DEGs were uniquely down-regulated and a large fraction of these genes—20 and 77% respectively—were uncharacterized protein. Among the remaining 6 DEGs with known biological functions, transcripts with annotation of succinate dehydrogenase, elongation factor 3, ABC transporter ATP-binding protein ARB1, chitinase, biphenyl-2,3-diol 1,2-dioxygenase and peptidyl-prolyl cis–trans isomerase NIMA-interacting 4 were identified (Table 4).

In recent years, there are many studies using transcriptome analysis to explore the response mechanism of fungi to DMI or other fungicides. These results indicated that expression of genes involved in sterol biosynthesis, cell wall integrity, MFS transporters, ATP-binding cassette (ABC) transporters and oxidative stress response were associated with fungicide resistance in multiple fungi^31,70–72^. In this paper, we found that MFS transporters, ABC transporter ATP-binding protein ARB1, chitinase and multiple oxidoreductases participated in the resistance regulation to phenamacril in Fo. The above mentioned data represent the first report of response to phenamacril in Fo and contribute to our knowledge in the mechanisms associated with fungicide resistance and fungicidal activity development in this fungal species complex.

## Conclusion

In this study, we found that the amino acid substitutions (V151A and S418T) in FoMyo5 cause natural low resistance to phenamacril in the plant pathogenic Fo isolates. By a comparative transcriptome analysis of phenamacril-resistant and -sensitive isolates after 1 μg/mL of phenamacril treatment, a series of DEGs that might be associated with resistance regulation and fungicidal activity of phenamacril were identified. These genes were involved in oxidation–reduction processes, MFS transporters, ATP-dependent RNA helicase and ribosomal biogenesis related proteins. These results indicated that phenamacril not only seriously affected the cytoskeletal protein binding and ATPase activity of sensitive isolate, but also suppressed ribosome biogenesis in all the isolates. This study provides deeper insight into resistance regulation mechanism and fungicidal activity of phenamacril in Fo and reference for the development of new fungicides to control diseases caused by Fo.

## Methods

### Isolates, chemicals and culture conditions

The isolates used in this study are listed in Table S1 and included the Fo isolate Fo3_a from cornea of patients with fungal keratitis and seven other Fo formae speciales LA0, CAO0, Fo1st, FoII5, Fo3-2, FoX-KW and FoHGKW from the roots of chili pepper, strawberry, lotus, banana, eggplant, watermelon and cucumber, respectively. All the isolates were routinely maintained at 28 °C on Difco™ Potato Dextrose Agar plates (PDA, suspend 39 g of the powder in 1 L of purified water and autoclave at 121 °C for 15 min). For mycelial growth assays, the isolates were grown at 28 °C on PDA plates for 7 days. For sporulation assays, 15 fresh mycelial plugs taken from the periphery of a 3-day-old colony of Fo isolates Fo1st, FoII5 and Fo3_a were added to a 250-mLflask containing 150 mL of Difco™ Potato Dextrose Broth (PDB, suspend 24 g of the powder in 1 L of purified water and autoclave at 121 °C for 15 min) medium.

Technical-grade phenamacril (95%; experimental code JS399-19), which was kindly provided by the Jiangsu Pesticide Research Institute Co., Ltd, Nanjing, China, was dissolved in methanol to 10 mg/mL and stored at 4 °C.

### Fungicide susceptibility testing

Phenamacril was added into autoclaved PDA media for testing inhibition of mycelia growth. Mycelial plugs (5 mm in diameter) taken from the margin of a 3-day-old colony were placed on the center of PDA plates amended with phenamacril at: 0, 0.2, 0.4, 0.8, or 1.6 μg/mL for sensitive isolates; 0, 2, 4, 8,or 16 μg/mL for Fo resistant isolates determined by EC_50_ values as the previous classification methods^25^. Three replicates for each concentration were used for each isolate. After cultures were kept at 28 °C for 7 days, the colonies were photographed and colony diameters were measured; the diameter (5 mm) of the original mycelial plugs were subtracted from each measurement. The 50% effective concentration (EC_50_) values of strains were calculated by regressing percentage growth inhibition against the log of fungicide concentration with DPS v9.01 software (Hangzhou Reifeng Information Technology Ltd., Hangzhou, China). Each experiment yields a set of EC_50_s and the experiment was performed three times.

### Sequence alignment of FoMyo5 motor domains

All Fo isolates were cultured in PDB at 28 °C for 3 days and the mycelia were collected and finely ground to a powder using a mortar and pestle with liquid nitrogen. The total RNA was extracted using the E.Z.N.A. Fungal RNA Kit (Omega Bio-tek, Inc., Norcross, USA) following the manufacturer’s instructions and used for reverse transcription with the PrimeScript™ RT reagent Kit (TaKaRa).The sequences of FoMyo5 motor domains were amplified from the cDNAs of all Fo isolates using the primer pairs FoMyo5F/FoMyo5R in this study (FoMyo5F:5’-ATGGGAATATCAAGACGC-3’; FoMyo5R: 5’-TTTGATAAAGGCCTTGGT-3’), which were synthesized by Tsingke Biotechnology Co., Ltd. Then these amplicons were gel purified using the OMEGA BIO-TEK (Shanghai, China) gel purification kit, cloned into the PMD18-T vector and sequenced in Tsingke Biotechnology Co., Ltd. And we aligned these FoMyo5 motor domains using Bioedit 7.2 software (Isis Pharmaceuticals).

### Sampling for RNA extraction

The spores harvested from 3-day-old PDB cultures of one phenamacril-sensitive Fo isolate Fo3_a and two phenamacril-resistant Fo isolates FoII5 and Fo1st were collected^24^ and suspended in sterile distilled water at 1 × 10^6^ spores/mL. The freshly harvested spores of each isolate were cultured in two flasks containing 100 mL liquid YEPD medium (w/v, 1% peptone, 0.3% yeast extract, 2% glucose) on a shaking table at a speed of 175 r/min for 12 h in the dark at 28 °C and each flask were inoculated with 100 μL of the spore suspensions. Then we added 10 μL 10 mg/mL phenamacril into the flasks of treatment groups and made the final concentration of phenamacril 1 μg/mL. we added 10 μL methanol into the flasks of control groups (CK) and made the final concentration of methanol 0.1 μL/mL. A total of 3 control isolates and 3 treatment isolates continued to be cultured for 12 h at 28 °C. After 24 h, the young mycelium were collected and finely ground to a powder using a mortar and pestle with liquid nitrogen and the total RNA was extracted using the above method. The experiment was repeated three times and we got a total of 18 RNA samples.

### RNA‑seq libraries construction and Illumina sequencing

The RNA purity was detected with a NanoDrop 2000 spectrophotometer (Thermo Scientific, Waltham, MA, USA) and RNA integrity was assessed using the RNA Nano 6000 Assay Kit of the Bioanalyzer 2100 system (Agilent Technologies, CA, USA). All samples passed the quality screening steps and were used for the subsequent steps. Total RNA was used as input material for the RNA libraries preparations. Briefly, mRNA was purified from total RNA using poly-T oligo-attached magnetic beads. Fragmentation was carried out using divalent cations under elevated temperature in First Strand Synthesis Reaction Buffer(5X). First strand cDNA was synthesized using random hexamer primer and M-MuLV Reverse Transcriptase, then use RNaseH to degrade the RNA.Second strand cDNA synthesis was subsequently performed using DNA Polymerase I and dNTP. Remaining overhangs were converted into blunt ends via exonuclease/polymerase activities. After adenylation of 3’ ends of DNA fragments, Adaptor with hairpin loop structure were ligated to prepare for hybridization. In order to select cDNA fragments of preferentially 370–420 bp in length, the library fragments were purified with AMPure XP system (Beckman Coulter, Beverly, USA). Then PCR was performed with Phusion High-Fidelity DNA polymerase, Universal PCR primers and Index (X) Primer. At last, PCR products were purified (AMPure XP system) and library quality was assessed on the Agilent Bioanalyzer 2100 system. After quantity and quality monitoring, 18 cDNA libraries were sequenced using the Illumina Novaseq 6000 platform in the Novogene Corporation at Bejing, China.

### Fo de novo transcriptome assembly and analysis

Raw reads of fastq format were firstly processed through in-house perl scripts. In this step, clean reads were obtained for the 18 libraries by removing reads containing adapter, reads containing N base and low quality reads from raw data with Trimmomatic^73^. At the same time, Q20, Q30 and GC content the clean data were calculated. All the downstream analyses were based on the clean data with high quality. Hisat2 v2.0.5 was used to align the paired-end clean reads to the reference genome of *Fusarium odoratissimum* NRRL 54006 reference genome (GCF_000260195.1, FO_II5_V1—Genome—Assembly—NCBI (nih.gov)) based on the gene model annotation file^74^. The mapped reads of each sample were assembled by StringTie (v1.3.3b) in a reference-based approach^75^. StringTie uses a novel network flow algorithm as well as an optional de novo assembly step to assemble and quantitate fulllength transcripts representing multiple splice variants for each gene locus. featureCounts v1.5.0-p3 was used to count the reads numbers mapped to each gene^76^. And then FPKM of each gene was calculated based on the length of the gene and reads count mapped to this gene. FPKM, expected number of Fragments Per Kilobase of transcript sequence per Millions base pairs sequenced, considers the effect of sequencing depth and gene length for the reads count at the same time, and is currently the most commonly used method for estimating gene expression levels.

### Identification of differential expression genes (DEGs) and clustering analysis

Differential genes expression level of the three isolates under 1 μg/mL phenamacril treatment and 0.1 μL /mL methanol treatment conditions were compared by the Wald test using the DESeq2 R package (1.20.0). The comparison groups include Fo3_a vs. Fo3_aCK, Fo1st vs. Fo1stCK and FoII5 vs. FoII5CK, that CK represents the control groups treated with 0.1 μL/mL methanol and others represent the treatment groups treated with 1 μg/mL phenamacril. DESeq2 provide statistical routines for determining differential expression in digital gene expression data using a model based on the negative binomial distribution. The resulting P-values were adjusted using the Benjamini and Hochberg’s approach for controlling the false discovery rate . Genes with an adjusted *P*-value < 0.05 and |log2 (Fold change)|> 1 were assigned as significantly differentially expressed^77,78^. Identification of unique or overlapping genes within the DEG datasets and the generation of Venn diagrams were determined using Venny 2.1 https://bioinfogp.cnb.csic.es/tools/venny/index.html.

The differential genes of all comparison groups were taken and collected as the differential gene set. We used H-cluster method to cluster the expression of differential genes after normalization with log_2_ (FPKM + 1). We plot the heatmap and genes or samples with similar expression patterns in the heatmap are gathered together.

### GO, KEGG and PPI analysis of differentially expressed genes

Gene Ontology (GO) enrichment analysis of differentially expressed genes was implemented by the R (4.1.1) and clusterProfiler R package (v4.0.5), in which gene length bias was corrected. GO terms with corrected *P*-value less than 0.05 were considered significantly enriched by differential expressed genes^79^. Kyoto Encyclopedia of Genes and Genomes (KEGG) is a database resource for understanding high-level functions and utilities of the biological system, such as the cell, the organism and the ecosystem, from molecular-level information, especially large-scale molecular datasets generated by genome sequencing and other high-through put experimental technologies (http://www.genome.jp/kegg/). We used cluster Profiler R package to test the statistical enrichment of differential expression genes in KEGG pathways^80,81^.

Protein Protein Interaction network (PPI) analysis of differentially expressed genes was based on the STRING database, which known and predicted Protein–Protein Interactions^82^. We provide differential gene protein interaction network data file, which can be directly imported into Cytoscape software for visual editing. For example, the size of nodes in the interaction network diagram is directly proportional to the degree of this node, that is, the more edges connected to this node, the greater its degree, the larger the nodes, and these nodes may be in a more core position in the network. Node1_protein and node2_protein represents interacting protein, as well as node1_gene and node2_gene represents the gene ID corresponding to the interacting protein. The score indicates the degree of interaction.

## Supplementary Information

Supplementary Information.

## Data Availability

Isolates of *Fusarium oxysporum* used in this study are available upon request. The raw sequence data from the 18 samples reported in this paper have been deposited in the Genome Sequence Archive (Genomics, Proteomics & Bioinformatics 2022) in National Genomics Data Center (Nucleic Acids Res 2022), China National Center for Bioinformation / Beijing Institute of Genomics, Chinese Academy of Sciences (GSA: CRA006003) that are publicly accessible at https://ngdc.cncb.ac.cn/gsa. The data and material that support the findings of this study are available from the corresponding author on request.
