# Supplementary material for: Comparative transcriptome analysis reveals the resistance regulation mechanism and fungicidal activity of the fungicide phenamacril in Fusarium oxysporum
Source: Sci Rep. 2022 Jun 30;12:11081. doi: 10.1038/s41598-022-15188-5 (PMC9247061; doi:10.1038/s41598-022-15188-5)
Supplement: Supplementary file 1 — Supplementary Information. [file 41598_2022_15188_MOESM1_ESM.docx]

**Supplementary Information**

**Comparative Transcriptome Analysis Reveals the Resistance Regulation Mechanism and Fungicidal activity of the Fungicide Phenamacril in *Fusarium oxysporum***

**Zhitian Zheng^a,^** **^*^, Huaqi Liu^a^, Yunyong Shi^a^, Zao Liu^a^, Hui Teng^a^, Sheng Deng^b,*^, Lihui Wei^b^, Yunpeng Wang^a,*^ , Feng Zhang^c^**

**^a^**School of Life Science and Food Engineering, Huaiyin Institute of Technology, Huai’an 223003, China

**^b^**Institute of Plant Protection, Key Lab of Food Quality and Safety of Jiangsu Province-State, Jiangsu Academy of Agricultural Sciences, Nanjing, 210014, Peoples Republic of China.

**^c^**Key Laboratory of Pesticide, College of Plant Protection, Nanjing Agricultural University, Nanjing 210095, China,

**^*^Corresponding author:** *E-mail addresses:*zztsdta@yeah.net (Z. Z.), dengsheng@jaas.ac.cn (S. D.), ypwang@hyit.edu.cn (Y. W.)

**Fig. S1. Colony morphology of *Fusarium oxysporum* isolates.** Colony morphology of Fo3-2, LA0, FoII5, Fo1st, CAO0, Fo3_a, FoX-KW and FoHGKW. Isolates were grown on solid media (PDA) for 7 days at 28 °C.


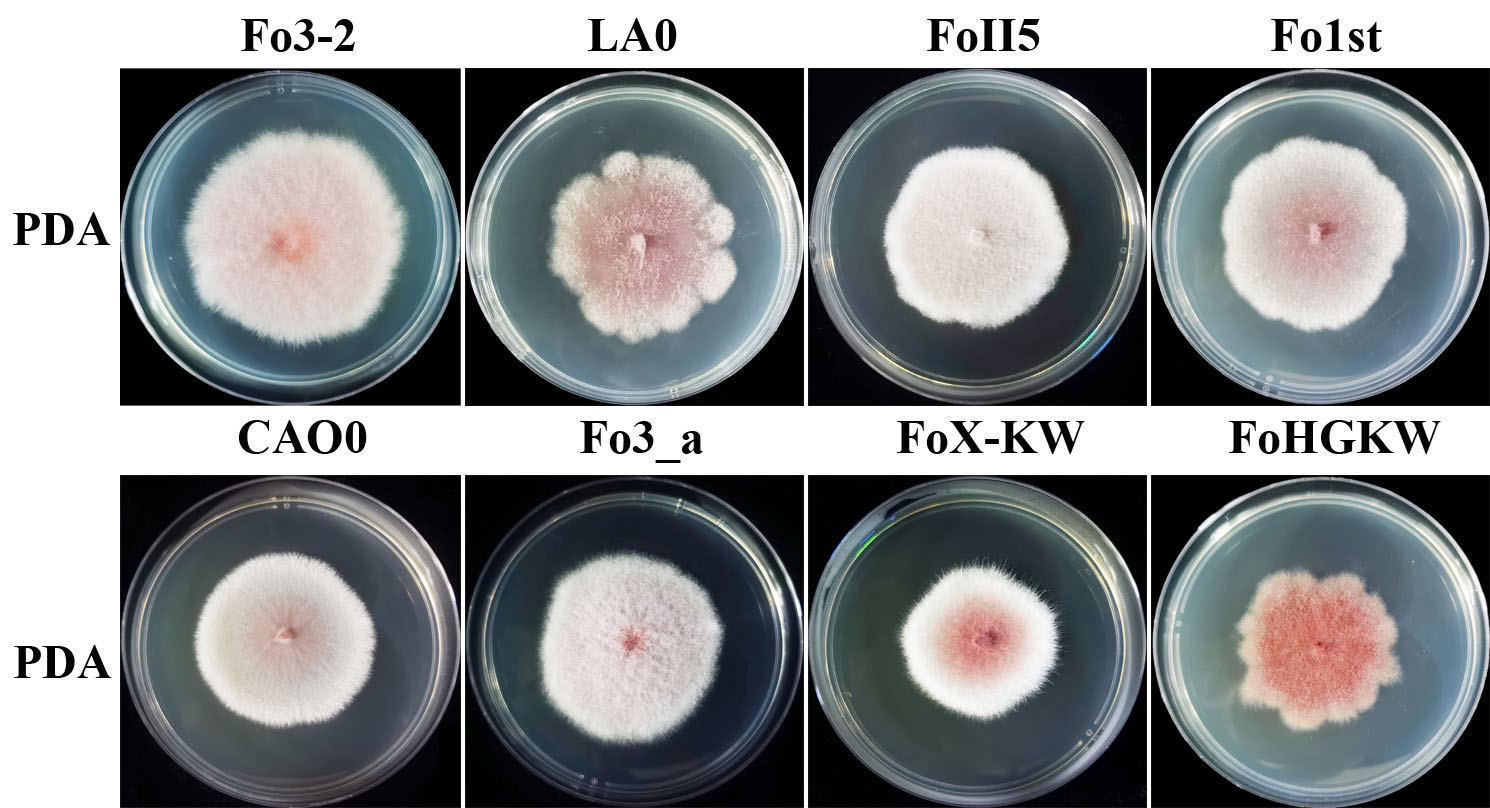


**Fig. S2.** **Boxplot of the log transformed FPKM expression distribution across six samples.** Each sample is represented by boxes of different colors and contains three biological replicates, represented by the numbers 1, 2, 3. CK represents the control groups and others represent the treatment groups with 1μg/mL phenamacril. FPKM: expected number of fragments per kilobase of transcript sequence per millions base pairs sequenced. The solid horizontal line represents the median, and the box encompasses the lower and upper quartiles.


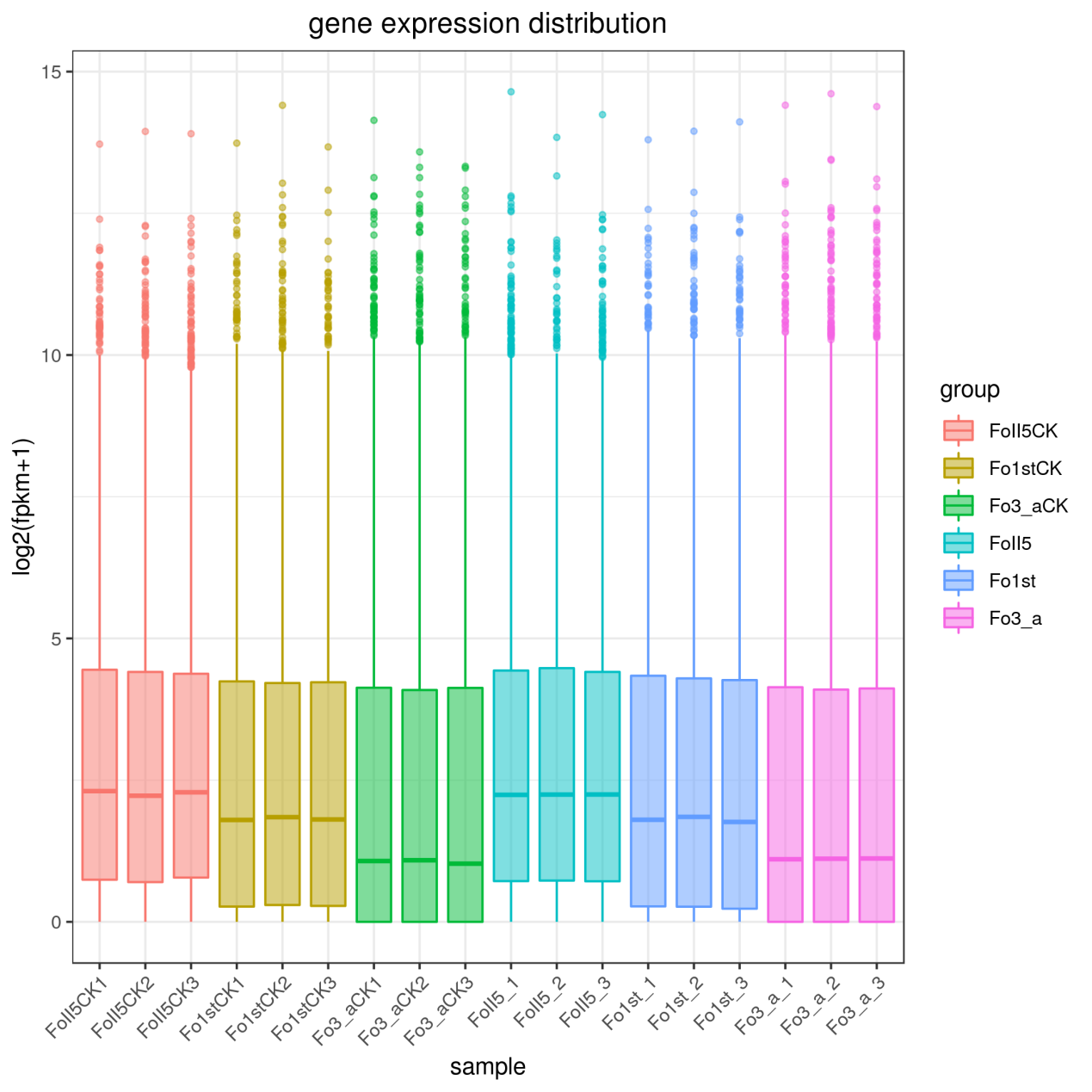


**Fig. S3. Hierarchical clustering and heatmap of DEGs based on FPKM.** The abscissa represents the sample name and the ordinate represents the FPKM values normalized with log_2_ (FPKM+1) of DEGs. Genes in red and blue represent significantly up-regulated or down-regulated expression, respectively. The darker the color, the higher the expression level. CK represents the control groups and others represent the treatment groups with 1μg/mL phenamacril.


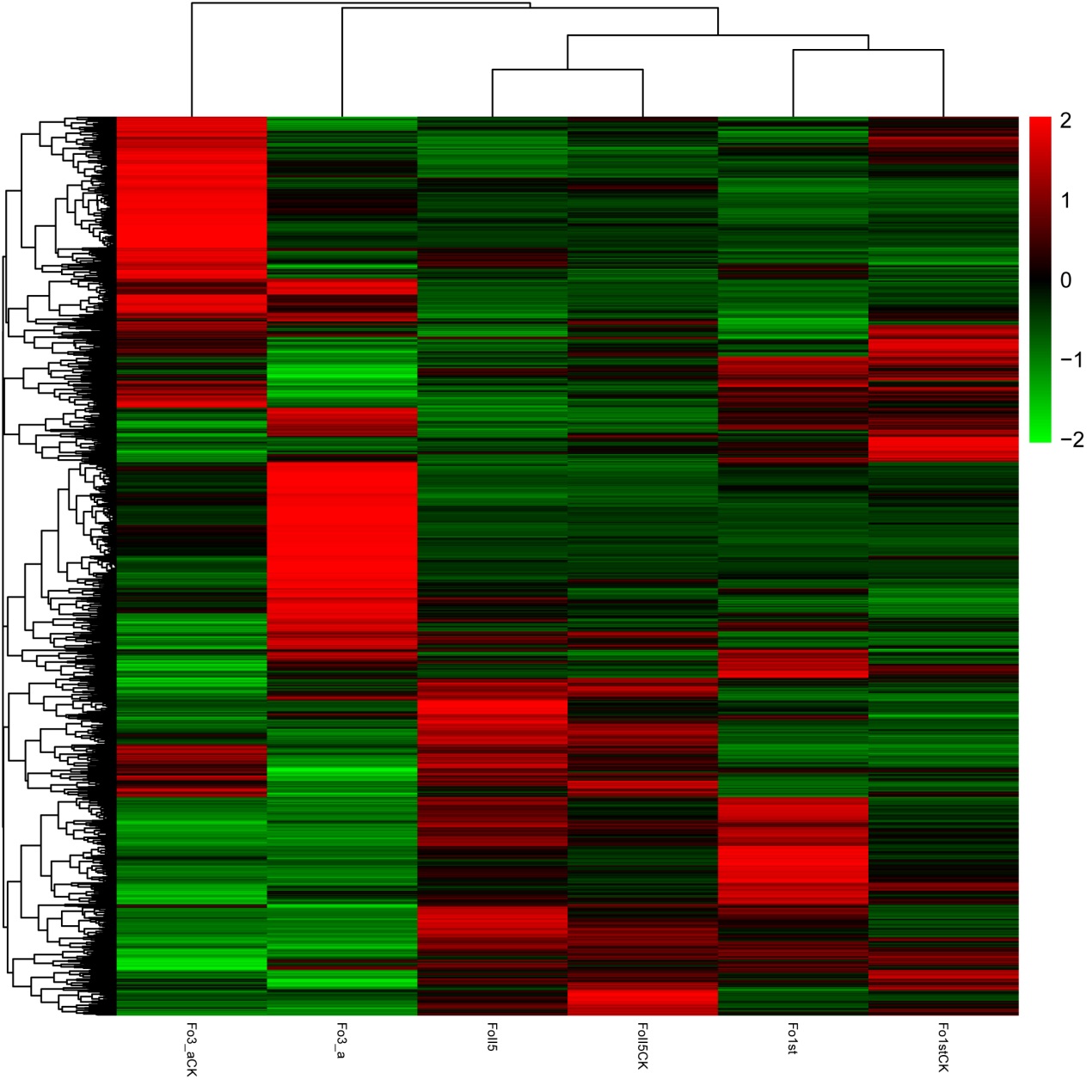


Table S1. *F. oxysporum* formae specialis used in this study.

| **Isolates** | **Description** |
| --- | --- |
| Fo3_a | *Fusarium oxysporum* strain, isolated from cornea of patients with fungal keratitis |
| LA0 | *Fusarium oxysporum* f. sp. *capsicum*, isolated from the chili pepper plant of Capsicum wilt |
| CAO0 | *Fusarium oxysporum* f. sp. *fragariae*, isolated from the strawberry plant of Fusarium wilt |
| Fo1st | *Fusarium oxysporum* f. sp. *nelumbicola* , isolated from the root of Lotus wilt |
| FoII5 | *F. oxysporum* f. sp*. cubense* race 4, isolate code NRRL54006 |
| Fo3-2 | *Fusarium oxysporum* f. sp. *melongenae*, isolated from the eggplant of Eggplant wilt |
| FoX-KW | *Fusarium oxysporum* f. sp. *niveum*, isolated from the watermelon of Watermelon wilt |
| FoHGKW | *Fusarium oxysporum* f. sp. *cucumerinum*, isolated from the cucumber of Cucumber wilt |

Table S2. Proteins that interact directly or indirectly with FoMyo5 in Fo3_a vs. Fo3_aCK comparison group.

| Gene id | Gene name | Fo3_a vs. Fo3_aCK | | Annotation |
| --- | --- | --- | --- | --- |
|  |  | Fold change | R^a^ |  |
| 42025702 | FOIG_00527 | 1.53 | up | Cortactin |
| 42025582 | FOIG_00407 | 2.83 | up | FoMyo5 |
| 42032111 | FOIG_06936 | 2.68 | up | Fimbrin |
| 42032886 | FOIG_07711 | 1.38 | up | Actin-like protein 3 |
| 42033493 | FOIG_08318 | 1.50 | up | Actin cytoskeleton-regulatory complex protein END3 |
| 42029775 | FOIG_04600 | 1.63 | up | Actin-like protein 2 |

^a^ up or down-regulation. CK represents the control groups and others represent the treatment groups with 1μg/mL phenamacril.

Table S3. The annotated KEGG metabolic pathways in the Fo1st vs. Fo1stCK and FoII5 vs. FoII5CK comparison groups.

| **Fo1 st vs. Fo1stCK^a^** | | **FoII5 vs. FoII5CK** | |
| --- | --- | --- | --- |
| **KEGGID** | **Description** | **KEGGID** | **Description** |
| fox04146 | Peroxisome | fox00910 | Nitrogen metabolism |
| fox03008 | Ribosome biogenesis in eukaryotes | fox03008 | Ribosome biogenesis in eukaryotes |
| fox01110 | Biosynthesis of secondary metabolites | fox00010 | Glycolysis / Gluconeogenesis |
| fox01212 | Fatty acid metabolism | fox00650 | Butanoate metabolism |
| fox00071 | Fatty acid degradation | fox00100 | Steroid biosynthesis |
| fox00630 | Glyoxylate and dicarboxylate metabolism | fox00410 | beta-Alanine metabolism |
| fox00280 | Valine, leucine and isoleucine degradation | fox00051 | Fructose and mannose metabolism |
| fox00260 | Glycine, serine and threonine metabolism | fox04146 | Peroxisome |
| fox01210 | 2-Oxocarboxylic acid metabolism | fox00450 | Selenocompound metabolism |
| fox01230 | Biosynthesis of amino acids | fox00280 | Valine, leucine and isoleucine degradation |
| fox01200 | Carbon metabolism | fox01040 | Biosynthesis of unsaturated fatty acids |
| fox00061 | Fatty acid biosynthesis | fox00190 | Oxidative phosphorylation |
| fox00330 | Arginine and proline metabolism | fox00565 | Ether lipid metabolism |
| fox01040 | Biosynthesis of unsaturated fatty acids | fox00860 | Porphyrin and chlorophyll metabolism |
| fox00410 | beta-Alanine metabolism | fox00130 | Ubiquinone and other terpenoid-quinone biosynthesis |
| fox00310 | Lysine degradation | fox04136 | Autophagy - other |
| fox00020 | Citrate cycle (TCA cycle) | fox04144 | Endocytosis |
| fox00460 | Cyanoamino acid metabolism | fox00290 | Valine, leucine and isoleucine biosynthesis |
| fox00340 | Histidine metabolism | fox00770 | Pantothenate and CoA biosynthesis |
| fox00290 | Valine, leucine and isoleucine biosynthesis | fox00350 | Tyrosine metabolism |
| fox00770 | Pantothenate and CoA biosynthesis | fox00030 | Pentose phosphate pathway |
| fox00620 | Pyruvate metabolism | fox00680 | Methane metabolism |
| fox00910 | Nitrogen metabolism | fox00260 | Glycine, serine and threonine metabolism |
| fox03020 | RNA polymerase | fox04070 | Phosphatidylinositol signaling system |
| fox00650 | Butanoate metabolism | fox04145 | Phagosome |
| fox00350 | Tyrosine metabolism | fox00640 | Propanoate metabolism |
| fox00130 | Ubiquinone and other terpenoid-quinone biosynthesis | fox00561 | Glycerolipid metabolism |
| fox00100 | Steroid biosynthesis | fox00562 | Inositol phosphate metabolism |
| fox00380 | Tryptophan metabolism | fox00310 | Lysine degradation |
| fox02010 | ABC transporters | fox00071 | Fatty acid degradation |
| fox00860 | Porphyrin and chlorophyll metabolism | fox00040 | Pentose and glucuronate interconversions |
| fox00400 | Phenylalanine, tyrosine and tryptophan biosynthesis | fox01212 | Fatty acid metabolism |
| fox00030 | Pentose phosphate pathway | fox00250 | Alanine, aspartate and glutamate metabolism |
| fox00250 | Alanine, aspartate and glutamate metabolism | fox01210 | 2-Oxocarboxylic acid metabolism |
| fox00760 | Nicotinate and nicotinamide metabolism | fox00620 | Pyruvate metabolism |
| fox00790 | Folate biosynthesis | fox04120 | Ubiquitin mediated proteolysis |
| fox00640 | Propanoate metabolism | fox00630 | Glyoxylate and dicarboxylate metabolism |
| fox00360 | Phenylalanine metabolism | fox00360 | Phenylalanine metabolism |
| fox03018 | RNA degradation | fox00564 | Glycerophospholipid metabolism |
| fox00040 | Pentose and glucuronate interconversions | fox00270 | Cysteine and methionine metabolism |
| fox00053 | Ascorbate and aldarate metabolism | fox01200 | Carbon metabolism |
| fox00670 | One carbon pool by folate | fox04138 | Autophagy - yeast |
| fox00220 | Arginine biosynthesis | fox00380 | Tryptophan metabolism |
| fox00520 | Amino sugar and nucleotide sugar metabolism | fox00520 | Amino sugar and nucleotide sugar metabolism |
| fox00300 | Lysine biosynthesis | fox01230 | Biosynthesis of amino acids |
| fox00970 | Aminoacyl-tRNA biosynthesis | fox04011 | MAPK signaling pathway - yeast |
| fox00480 | Glutathione metabolism | fox04141 | Protein processing in endoplasmic reticulum |
| fox00500 | Starch and sucrose metabolism | fox03013 | RNA transport |
| fox04136 | Autophagy - other | fox03040 | Spliceosome |
| fox00730 | Thiamine metabolism | fox03010 | Ribosome |
| fox03450 | Non-homologous end-joining |  |  |
| fox00564 | Glycerophospholipid metabolism |  |  |
| fox00511 | Other glycan degradation |  |  |
| fox00780 | Biotin metabolism |  |  |
| fox00052 | Galactose metabolism |  |  |
| fox04139 | Mitophagy - yeast |  |  |
| fox03040 | Spliceosome |  |  |
| fox00565 | Ether lipid metabolism |  |  |
| fox03440 | Homologous recombination |  |  |
| fox00270 | Cysteine and methionine metabolism |  |  |
| fox00230 | Purine metabolism |  |  |
| fox03410 | Base excision repair |  |  |
| fox00563 | Glycosylphosphatidylinositol (GPI)-anchor biosynthesis |  |  |
| fox00010 | Glycolysis / Gluconeogenesis |  |  |
| fox00680 | Methane metabolism |  |  |
| fox00190 | Oxidative phosphorylation |  |  |
| fox00561 | Glycerolipid metabolism |  |  |
| fox04138 | Autophagy - yeast |  |  |
| fox04011 | MAPK signaling pathway - yeast |  |  |
| fox00562 | Inositol phosphate metabolism |  |  |
| fox00510 | N-Glycan biosynthesis |  |  |
| fox03420 | Nucleotide excision repair |  |  |
| fox03015 | mRNA surveillance pathway |  |  |
| fox04141 | Protein processing in endoplasmic reticulum |  |  |
| fox03013 | RNA transport |  |  |
| fox03010 | Ribosome |  |  |

^a^CK represents the control groups and others represent the treatment groups with 1μg/mL phenamacril.
